# Partial substitution of soybean meal with microalgae meal (*Arthrospira spp.* – Spirulina) in grower and finisher diets for broiler chickens: implications on performance parameters, footpad dermatitis occurrence, breast meat quality traits, amino acid digestibility and plasma metabolomics profile

**DOI:** 10.1016/j.psj.2024.103856

**Published:** 2024-05-21

**Authors:** Marco Zampiga, Luca Laghi, Francesca Soglia, Raffaela Piscitelli, Jonathan Dayan, Massimiliano Petracci, Alessio Bonaldo, Federico Sirri

**Affiliations:** ⁎ Department of Agricultural and Food Sciences, Alma Mater Studiorum - University of Bologna, Ozzano dell'Emilia, Bologna 40064, Italy; † Department of Animal Science, The Robert H. Smith Faculty of Agriculture, Food and Environment, The Hebrew University of Jerusalem, Rehovot 7610001, Israel; ‡ Department of Veterinary Medical Sciences, Alma Mater Studiorum - University of Bologna, Ozzano Emilia, Bologna 40064, Italy

**Keywords:** broiler chicken, microalgae meal, productive performance, meat quality, metabolomic

## Abstract

This trial was conducted to evaluate the effects of replacing soybean meal with microalgae meal (**MM**; *Arthrospira spp.*) during grower and finisher phases on productive performance, footpad dermatitis (**FPD**) occurrence, breast meat quality, amino acid digestibility and plasma metabolomics profile of broiler chickens. One thousand day-old Ross 308 male chicks were divided into 5 experimental groups (8 replicates, 25 birds/each): **CON**, fed a commercial soybean-based diet throughout the trial (0–41 d); **F3** and **F6**, fed the CON diet up to 28 d of age and then a finisher diet (29–41 d) with either 30 or 60 g MM/kg, respectively; and **GF3** and **GF6**, receiving CON diet until 14 d and then diets containing 30 or 60 g MM/kg from 15 to 41 d, respectively. All diets were iso-energetic and with a similar amino acid profile. Growth performances were recorded on a pen basis at the end of each feeding phase and apparent ileal amino acid digestibility was determined at 41 d. Footpad dermatitis occurrence was assessed on all processed birds, while breast and plasma samples were collected for meat quality and metabolomics analysis (proton nuclear magnetic resonance - **^1^H-NMR**). At 41 d, CON group showed higher body weight than F6 and GF6 ones (2,541 vs. 2,412 vs. 2,384 g, respectively; *P* < 0.05). Overall, GF6 group exhibited the highest feed conversion ratio, while F3 did not present significant differences compared to CON (1.785 vs. 1.810 vs. 1.934 g feed/g gain, respectively for CON, F3 and GF6; *P* < 0.01). The occurrence and the risk of developing FPD were similar among groups. MM administration increased breast meat yellowness and reduced amino acid digestibility (*P* < 0.001). The ^1^H-NMR analysis revealed variations in the levels of some circulating metabolites, including histidine, arginine and creatine, which play important metabolic roles. Overall, these findings can contribute to expand the knowledge about the use of *Arthrospira spp.* as protein source in broiler diets.

## INTRODUCTION

Because of their nutritional value and market price, oilseed meals such as soybean meal (**SBM**) are broadly used worldwide as feed protein raw materials especially in farmed monogastric animals (FEFAC, 2022). In Europe, however, the widespread cultivation of protein crops like soybean is limited by sub-optimal climatic conditions, resulting in a substantial feed protein deficit that is covered through imports from third countries such as United States, Brazil and Argentina (Kim et al., 2019; FAO, 2021). A recent report has shown that EU average self-sufficiency rate for high-protein feed materials is lower than 30%, with more than 95% of SBM used in the “old continent” that has an extra-EU origin (FEFAC, 2022). Similarly, China ranked first in the list of top soybean importer countries accounting for approximately 60% of the global soybean trade (De Maria et al., 2020), with imports rates that have soared exponentially over the last 20 y (i.e., +200, 700, and 2,000 from Argentina, United States and Brazil, respectively; Fuchs et al., 2019). In recent times, the overall sustainability of such process has been a matter of debate because of its environmental implications at both local and global levels (De Maria et al., 2020). In South America, where more than half of world's soybean is currently produced (FAO, 2021), the area destined to soybean cultivation has more than doubled in the first 2 decades of 2000s (from 26.4 Mha in 2001 to 55.1 Mha in 2019) mostly at the expenses of pastures and natural ecosystems, generating ecological and environmental issues such as soil erosion, deforestation, land use changes and biodiversity losses (Song et al., 2021). In addition, soybean transportation over long distances (e.g., from US and South America to Europe or China) has environmental consequences due to fossil fuel use and greenhouse gas emissions (da Silva et al., 2010). On the opposite, importing countries can face waste problems due to nutrients overload and alteration of their natural cycles (Taelman et al., 2015) as well as socio-economic issues since the dependency on the international supply can potentially undermine the resilience and sustainability of the local livestock sector (Elshamy and Rösch, 2022).

Fraanje and Garnett (2020) reported that large part of soybean production worldwide is fated to livestock feeding, with poultry contributing to approximately 37% of total soybean consumption. Indeed, SBM is considered the protein source *par excellence* for poultry feed manufacturing mostly because of its high crude protein content and balanced essential amino acid profile (Beski et al., 2015). Although poultry production has a relatively low emission intensity (Mottet and Tempio, 2017), the large use of soybean (mainly as SBM) could represent an environmental sustainability bottleneck for this sector, particularly in light of the expected increase in poultry meat demand supported by the demographic growth and the consumers’ appreciation for this type of meat (OECD/FAO, 2023). For these reasons, the identification of alternative, more sustainable protein feedstuff that might substitute SBM in poultry diets while maintaining animal performance, health and product quality has taken center stage in recent years.

Microalgae such as *Arthrospira spp.* (Spirulina) can be considered as a promising SBM-alternative to be used in poultry diets (Saadaoui et al., 2021). Indeed, these photosynthetic aquatic microorganisms are able to produce protein-rich biomass with limited land utilization (either arable or marginal land) and with greater efficiency in CO_2_ fixation, absorption of solar energy radiation and nutrient uptake (including water, nitrogen, and phosphorous) compared to terrestrial plants, resulting in faster growth (Wang et al., 2008; Taelman et al., 2015). Although it has been demonstrated that microalgae can be safely administered in chicken diets (Świątkiewicz et al., 2015), their large-scale use as feed protein raw material is currently hindered by an uncompetitive market price and a scarce knowledge regarding the optimal dietary inclusion rate and nutrients digestibility (Saadaoui et al., 2021). In their review paper, Coudert et al. (2020) indicated that a dietary inclusion of microalgae around 20 g/kg seems to be suitable for broiler chickens, yet pointing out that only few studies have considered microalgae as a major source of dietary proteins. In our previous investigation (Zampiga et al., 2023), a significant impairment of broiler growth performance was observed when a dehydrated microalgae meal from geothermally cultivated, carbon neutral *Arthrospira spp.* (**MM**; Tzachor et al., 2022) was included at 50, 100, or 150 g/kg as partial replacement for soybean up to 22 d of age (i.e., starter and grower phases). According to these outcomes, the present trial was carried out to evaluate the effects of the partial replacement of SBM with moderate dosages of *Arthrospira spp.* meal (i.e., 30 and 60 g/kg) during the grower and finisher phases on productive performance, occurrence of footpad dermatitis (**FPD**), breast meat quality traits, apparent ileal amino acid digestibility and plasma metabolomics profile of broiler chickens.

## MATERIALS AND METHODS

### Ethic Statement

In the present research, which was evaluated and approved by the Ethical Committee of the University of Bologna (ID: 1145/2020), birds were raised, handled, and processed according to the current EU legislation (Directive 2007/43/EC; Regulation 2009/1099/EC; Directive 2010/63/EU).

### Animals and Housing

One thousand 1-day-old male Ross 308 chicks were supplied by a commercial hatchery. All chicks used for the study, which were obtained from the same breeder flock and hatching batch, were vaccinated at the hatchery against infectious bronchitis, Marek's disease, Newcastle and Gumboro diseases, and coccidiosis. Chicks were transported to an experimental, environmentally-controlled poultry facility and randomly allocated in 40 pens, whose concrete floor was covered with bedding material (i.e., wood shavings; 3–4 kg/m^2^). Each pen presented a circular pan feeder (ensuring 2 cm of front space/bird), an independent drinking system with 5 nipple-type drinkers (5 birds/nipple) and a labelled bin containing the feed to be administered to the birds. For each pen, the feeder was manually filled on a daily basis taking the feed from the corresponding bin. All equipment within the pen presented the same characteristics (i.e., type, dimension, and color). According to the EU legislation (Directive 2007/43/EC), the stocking density was always kept below 33 kg live-weight/m^2^ and the artificial photoperiod was defined according to the age of the birds: 23 h light to 1 h dark from 0 to 7 d and from 39 to 41 d, while 18 h light to 6 h dark (continuous) was adopted from 8 to 38 d. The environmental temperature was defined according to current recommendations based on bird age.

### Experimental Design and Diets

Up to 13 d of age, all birds received the same commercial starter feed mostly based on corn, wheat, and soybean (Table 1). Then, each pen was assigned to one of the following experimental groups according to a completely randomized block design with 8 replicate pens/group (25 birds/replicate): **CON** group, receiving a commercial diet with soybean (both full-fat and SBM) as main protein source during grower (14–28 d) and finisher phase (29–41 d); **F3** and **F6** groups, which were fed the CON diet during the grower phase and, for the finisher one, the CON diet with either 30 or 60 g MM/kg, respectively. Finally, the groups **GF3** and **GF6**, in which the MM was incorporated into the diet at either 30 or 60 g/kg during both grower and finisher phases (i.e., from 14 to 41 d of age). Each block (n = 8) was represented by a group of 5 adjacent pens in which each dietary treatment was represented once. The use of blocks was done to minimize any environmental effect potentially occurring in the poultry house. The analyzed chemical composition and amino acid profile of the MM (VAXA Impact Nutrition, Reykjavík, Iceland) were reported in our previous paper (Zampiga et al., 2023) and briefly summarized in the Supplementary Materials. All diets were formulated to meet nutritional recommendations (Aviagen, 2019), with analogous metabolizable energy content (i.e., iso-energetic diets) and with a similar amino acid profile, which was optimized by maintaining the same ratio of total essential amino acids to total lysine (Table 1). The inclusion of MM in the experimental diets was done mostly at the expenses of SBM, whereas the full-fat soybean content was not modified. Compared to CON diet, the inclusion of MM at 30 and 60 g/kg allowed a reduction in the overall amount of dietary soybean by about 16% and 33% in the grower phase and 20% and 40% in the finisher phase, respectively. All feeds were in a mash form and, as well as water, provided for ad-libitum consumption.

**Table 1:** Composition of the experimental diets.

	Starter (0–13 d)	Grower (14–28 d)			Finisher (29–41 d)		
Ingredients (g/kg)		CON-F3-F6	GF3	GF6	CON	F3-GF3	F6-GF6
Microalgae meal	0.00	0.00	30.0	60.0	0.00	30.0	60.0
Corn	424.1	448.5	482.3	516.4	417.7	451.7	485.2
Sorghum	0.00	0.00	0.00	0.00	50.0	50.0	50.0
Wheat	100.0	100.0	100.0	100.0	130.0	130.0	130.0
Vegetable oil	21.2	28.6	19.9	11.2	40.7	31.9	23.2
Wheat bran	20.0	20.0	20.0	20.0	20.0	20.0	20.0
Soybean meal	223.3	181.3	126.1	70.9	124.0	68.7	13.4
Full-fat soybean	100.0	149.9	149.9	149.9	150.0	150.0	150.0
Sunflower meal	20.0	20.0	20.0	20.0	20.0	20.0	20.0
Corn gluten	30.0	0.00	0.00	0.00	0.00	0.00	0.00
Pea	20.0	20.0	20.0	20.0	20.0	20.0	20.0
Calcium carbonate	3.70	5.50	6.10	6.70	9.10	9.70	10.4
Dicalcium phosphate	12.0	5.80	5.10	4.30	1.40	0.70	0.00
Sodium chloride	3.50	3.10	2.70	2.20	2.40	2.10	1.90
Sodium bicarbonate	0.60	0.70	0.60	0.50	1.70	1.30	1.00
Choline	1.00	1.00	1.00	1.00	1.00	1.00	1.00
Lysine sulphate (50%)	5.90	3.70	4.80	5.90	3.50	4.60	5.70
DL-Methionine (99%)	2.90	1.30	2.00	2.70	2.60	2.50	2.50
Met. Hydroxy-analogue	0.00	2.00	1.00	0.00	0.00	0.00	0.00
L-Threonine (98%)	1.30	0.90	0.90	0.80	0.80	0.80	0.70
Phytase	2.00	2.00	2.00	2.00	1.50	1.50	1.50
NSP enzyme	0.50	0.50	0.50	0.50	0.50	0.50	0.50
Amino acids mix (Arg+Val+Ile)	2.00	0.60	0.60	0.50	0.60	0.50	0.50
Mycotoxin binder	1.00	0.00	0.00	0.00	0.00	0.00	0.00
Vitamin-mineral premix^1^	5.00	4.50	4.50	4.50	2.50	2.50	2.50
**Composition (g/kg)**							
Dry Matter^2^	884.4	884.6	885.9	885.9	886.8	886.6	886.3
Crude protein^2^	228.1	208.8	206.7	204.5	187.8	185.6	183.5
Total lipid^2^	59.7	76.6	72.2	67.8	89.0	84.6	80.2
Crude fibre^2^	29.5	30.3	30.0	29.6	29.1	28.7	28.4
Ash^2^	51.3	46.4	44.9	43.5	42.7	41.2	39.7
Calcium (total)	7.60	6.50	6.50	6.50	6.10	6.10	6.10
Phosphorous (total)	6.00	4.80	4.80	4.80	3.90	3.90	3.90
Lysine (total)	14.0	12.6	12.6	12.6	11.0	11.0	11.0
Met + Cys (total)	10.5	9.70	9.70	9.70	8.70	8.70	8.70
Threonine (total)	9.50	8.50	8.50	8.50	7.50	7.50	7.50
AME (kcal/kg)	3,030	3,150	3,150	3,150	3,275	3,275	3,275

Abbreviations: AME, apparent metabolizable energy; NSP, non‐starch polysaccharides; CON, control; F3, 30 g/kg of microalgae meal during finisher phase; F6, 60 g/kg of microalgae meal during finisher phase; GF3, 30 g/kg of microalgae meal during grower and finisher phases; GF6, 60 g/kg of microalgae meal during grower and finisher phases.

1 Provided the following per kg of diet: vitamin A (retinyl acetate), 13,000 IU; vitamin D3 (cholecalciferol), 4,000 IU; vitamin E (DL-α_tocopheryl acetate), 80 IU; vitamin K (menadione sodium bisulfite), 3 mg; riboflavin, 6.0 mg; pantothenic acid, 6.0 mg; niacin, 20 mg; pyridoxine, 2 mg; folic acid, 0.5 mg; biotin, 0.10 mg; thiamine, 2.5 mg; vitamin B12 20 μg; Mn, 100 mg; Zn, 85 mg; Fe, 30 mg; Cu, 10 mg; I, 1.5 mg; Se, 0.2 mg; ethoxyquin, 100 mg.

2 Analyzed values.

### Productive Performance, Slaughtering Measurements and Meat Quality Traits

Birds were counted and weighed pen wise at placement (0 d), at each diet switch (13 and 28 d) and at slaughter (41 d). Similarly, feed consumption was determined on a pen basis at the end of each feeding phase (13, 28, and 41 d) as the difference between feed administered at the beginning of the phase and the residual at the end of it. On a daily basis, dead birds were registered and weighed to calculate mortality rate and to adjust performance data. Body weight (**BW**), daily weight gain (**DWG**), daily feed intake (**DFI**) and feed conversion ratio (**FCR**) were calculated for each feeding phase and for the overall trial period.

At 41 d, all birds were slaughtered in a commercial abattoir after being subjected to water-bath electrical stunning (200–220 mA, 1,500 Hz; Regulation 2009/1099/EC). The carcasses belonging to each group, which were clearly identified and kept separated from those of other groups, were mechanically processed and carcass and breast yields (without skin and bones) were determined on all birds on a treatment basis. One foot per bird was collected during processing to assess the presence of FPD, whose severity was scored as follows: 0 = no lesions; 1 = mild lesions, diameter < 0.8 cm; 2 = severe lesions, diameter > 0.8 cm or particularly diffused over the feet surface (Ekstrand et al., 1998).

After air chilling and deboning, 15 breast muscles (*Pectoralis major*) *per* groups were collected among those showing no visible defects (e.g., macroscopic evidence of muscle abnormalities, hemorrhages, or lesions) and transported under refrigerated conditions to the laboratories of the University of Bologna, where the main meat technological properties were evaluated as previously specified (Sirri et al., 2017). Briefly, meat ultimate pH (**pHu**) was measured 48 h *postmortem* with a portable pH-meter equipped with a stainless steel blade tip for meat penetration and temperature compensation (HI98163, Hanna Instruments Inc., Padova, Italy). The color profile (L* - lightness, a* - redness, b*- yellowness; CIE, 1978) was evaluated in triplicate on the medial surface (bone side) of the fillet by using a reflectance colorimeter (illuminant source C; Minolta Chroma Meter CR-400, Minolta Italia S.p.A., Milan, Italy). Water Holding Capacity was assessed by measuring drip and cooking losses. A parallelepiped meat cut (8 × 4 × 2 cm, weighing about 80 g) was excised from the cranial portion of each *P. major* muscle and stored at 4 ± 1°C in plastic boxes over sieved plastic racks. After 48 h, the excess of surface fluids was gently blotted, samples were weighed and drip loss calculated as percentage of weight lost. The same samples were vacuum packed and cooked in water bath until reaching an inner core temperature of 75°C. Then, the samples were chilled at room temperature and weighed to calculate cooking loss. The color profile of cooked samples was assessed as described above for fresh meat. Finally, subsamples (4 × 1 × 1 cm) were obtained from the cooked meat samples to determine the shear force. For this evaluation, a TA.HDi Heavy Duty texture analyzer (Stable Micro Systems Ltd., Godalming, Surrey, UK), equipped with a 5 kg loading cell and a Warner Bratzler shear probe, was used and the maximum force recorded when shearing the meat samples expressed as kg.

### Apparent Ileal Amino Acid Digestibility

From 35 to 41 d of bird age, the feed of CON, F3 and F6 groups was supplemented with titanium dioxide (TiO_2_; 3 g/kg), which was used as indigestible marker to evaluate apparent ileal amino acid (**AA**) digestibility at 41 d of age. The digestibility assay did not involve GF3 and GF6 groups to avoid potential biases due to the remarkably different BW at the beginning of the evaluation. Two birds/replicate pen, for a total of 16 birds *per* dietary treatment, were selected according to the average BW of each experimental group. Birds were humanely euthanized and ileum dissected from the Meckel's diverticulum to approx. 40 mm above the ileo-caecal junction to collect its content (Ravindran et al., 2017). Digesta were pooled, mixed, frozen at -20°C and then freeze-dried to obtain 3 pools of 8 g dried ileal content/group. Dried digesta and experimental diets samples were ground (0.5 mm sieve) and stored in airtight containers at −20°C until analyses. The amino acids concentration of feed and dried digesta samples was analyzed by AMINOLab (Evonik Industries, Hanau, Germany). The amount of titanium dioxide was determined through spectrophotometric analysis following the protocol established by Myers et al. (2004). Briefly, 0.5 gram of either feed or dried ileal content were placed into a glass tube with 13 ml of H_2_SO_4_ (96%; Carlo Erba Reagents s.r.l., Milano, Italy), 3.5 g of K_2_SO_4_ and 0.4 g of CuSO_4_ (Thompson & Capper Ltd, Runcorn, Cheshire, UK). A macro-Kjeldahl apparatus (Gerhardt Kjeldatherm; C. Gerhardt GmbH & Co. KG, Königswinter, Germany) was used for samples digestion at 420°C for 2 h. Then, 10 ml of H_2_O_2_ (30%; Carlo Erba Reagents s.r.l., Milano, Italy) were added to each tube and the total liquid weight was brought up to 100 g by adding distilled water. After filtration through Whatman n. 541, the aqueous phase was read at 410 nm with an UV/Vis Spectrophotometer (Jasco model 7800) previously calibrated using solutions containing 0, 2, 4, 6, 8, and 10 mg of TiO_2_. The digestibility coefficient of each AA was calculated according to the formula proposed by Ravindran et al. (2017):

$$Digestibility=1-\left[{\left(AA/TiO_{2}\right)}_{ileal}/{\left(AA/TiO_{2}\right)}_{diet}\right]$$

where *(AA / TiO_2_)_ileal_* and *(AA / TiO_2_)_diet_* represent the ratio between the concentration of the selected AA and the marker in the ileal digesta and in the diet, respectively. Results were expressed as percentage.

### Plasma Metabolomics Profile

At slaughtering (41 d), 16 broilers *per* group (i.e., 2 broilers/pen) were selected based on the average BW of each specific experimental group and used for blood sampling through wing vein withdrawal. Blood was collected into lithium-heparin vials and centrifuged to get plasma, which was conserved at −80°C for proton nuclear magnetic resonance (**^1^H-NMR**) spectrometry. For this analysis, which was carried out as described in our previous study (Zampiga et al., 2021), a ^1^H-NMR solution was produced with D_2_O, containing 10 mmol/L of 3-(trimethylsilyl)-propionic-2,2,3,3-d_4_ acid sodium salt (**TSP**) as a reference for NMR chemical shift and 2 mmol/L NaN_3_ to avoid microbial proliferation. Plasma samples were centrifuged (18,630 *× g* for 15 min at 4°C) and an aliquot of the supernatant (0.65 mL) was mixed with 0.1 mL of the ^1^H-NMR solution and centrifuged again at the same conditions. An AVANCE™ III spectrometer (Bruker, Milan, Italy) equipped with the Topspin software (v.3.5) was utilized for spectra recording. Temperature and frequency conditions were 298 K and 600.13 MHz, respectively. A CPMG-filter (400 echoes with a *τ* of 400 µs and a 180° pulse of 24 µs, for a total filter of 330 ms) was applied to suppress the signals from broad resonances originating from large molecules, whereas the water residual signal was suppressed through presaturation by employing the pulse sequence *cpmgpr1d* sequence. Each spectrum was collected by summing up 256 transients constituted by 32,000 data points encompassing a window of 7,184 Hz, divided by 5 s of relaxation delay. Topspin v3.5 was used to phase-adjust the spectra, which were subsequently exported to ASCII format through the script *convbin2asc* and imported into the R software environment employing in-house developed scripts. Signals were assigned to specific molecules by comparing their position in the spectra, shape and multiplicity with the Human Metabolome Database (Wishart et al., 2007) and Chenomx software libraries (Chenomx Inc., Edmonton, AB, Canada, v10), by relying on the routines made available by Chenomx software. TSP was considered as internal standard for the quantification of molecules concentration, while probabilistic quotient normalization (Dieterle et al., 2006) was applied to compensate for potential differences in water content among samples. As in Brugaletta et al. (2023), molecules concentration was determined according to the area of one of their signals, computed by the GSD (global spectra deconvolution) algorithm of MestReNova software (v14.2.0–26256; Mestrelab research S.L., Santiago De Compostela, Spain). A limit of quantification (**LOQ**) of 5 was considered. Before, a baseline adjustment was performed applying the Whittaker Smoother procedure and a line broadening of 0.3.

### Statistical Analysis

For all analyses, the dietary treatment was considered as the experimental factor and a significance level of *P* < 0.05 was defined. Performance data were analyzed by means of one-way blocked ANOVA with the replicate pen as experimental unit. Mortality data were arcsine transformed prior to analysis and the Tukey post-hoc test was applied for multiple comparisons among groups. In addition, contrasts were performed, when appropriate, to further explore the overall effects of MM inclusion (CON diet vs. MM diets), dosage (30 g MM/kg vs. 60 g MM/kg) and duration (MM in finisher phase vs. MM in grower and finisher phases). Slaughter yields were not statistically evaluated as data were collected on a group basis without replicates. Footpad dermatitis data were analyzed through the Pearson's Chi-squared test using the bird as experimental unit. In addition, FPD count data were also utilized for determining the incidence risk ratio. When the ratio was significant at 95% confidence interval, the risk of developing FPD was calculated as the risk ratio minus 1 and expressed as percentage (Brugaletta et al., 2022). For digestibility, meat quality and metabolomics insights, data were subjected to one-way ANOVA followed by Tukey post-hoc test. The experimental unit for the digestibility assay was the pool of freeze-dried ileal content, while the bird was considered for meat quality and metabolomics. Specifically, for the metabolomics analysis, the Box and Cox transformation (1964) was applied for normalizing molecule concentration data. Data obtained with the ^1^H-NMR analysis were further explored through robust Principal Component Analysis (**rPCA**; Hubert et al., 2005).

## RESULTS

### Growth Performance

At placement (0 d) and at the end of starter phase (13 d), broilers exhibited comparable BW (average group values ranging from 37.3 to 38.0 g and from 325 to 333 g, *P* = 0.21 and *P* = 0.90, respectively; Supplementary Materials). At 28 d (Table 2), GF6 groups showed significantly lower BW compared to CON group, with GF3 presenting an intermediate value (1,379 vs. 1,308 vs. 1,284 g/bird for CON, GF3 and GF6 groups, respectively; *P* < 0.001). From 14 to 28 d, DWG was lower in GF3 and GF6 groups than in CON group (69.6 vs. 64.6 vs. 62.5 g/bird/d for CON, GF3 and GF6, respectively; *P* < 0.001), while DFI was not affected by the dietary treatments. Both GF3 and GF6 groups exhibited greater FCR than CON group (1.636 vs. 1.777 vs. 1.811 g feed/g gain, respectively for CON, GF3 and GF6; *P* < 0.001). Mortality was not significantly different among groups. Overall, the contrasts analysis highlighted that the dietary administration of MM significantly impaired BW, DWG and FCR (1,396 vs. 1,296 g/bird, 70.8 vs. 63.5 g/bird/d, and 1,594 vs. 1.794 g feed/g gain, respectively; *P* < 0.01), while the dosage did not exert any relevant effect. As for the finisher phase (29–41 d), the significantly lowest DWG was shown by F6 birds (76.4 g/bird/d), followed by F3 ones (81.8 g/bird/d), while GF3 group was comparable to CON (87.5 vs. 89.5 g/bird/d, respectively). DFI was higher in GF3 and GF6 groups than in F6 group (176.1 vs. 171.5 vs. 166.9 vs. 180.3 vs. 181.0 g/bird/d, for CON, F3, F6, GF3 and GF6, respectively; *P* < 0.01). The F6 and GF6 groups showed higher FCR if compared to CON, whereas F3 and GF3 presented intermediate values (1.971 vs. 2.102 vs. 2.188 vs. 2.063 vs. 2.143 g feed/g gain, for CON, F3, F6, GF3 and GF6, respectively; *P* < 0.01). Mortality was not remarkably affected by the diet. In general, the dietary administration of MM had negative effects on performance traits (BW: 2,541 vs. 2,424 g/bird; DWG: 89.5 vs. 82.5 g/bird/d; FCR: 1.971 vs. 2.124 g feed/g gain, for CON and MM, respectively; *P* < 0.01). When the 2 dosages are compared, the incorporation of 60 g MM/kg significantly reduced DWG and increased FCR compared to 30 g/kg (80.5 vs. 84.6 g/bird/d and 2.165 vs. 2.083 g feed/g gain, respectively; *P* < 0.01). The duration of MM administration affected DWG and DFI, which were higher in birds receiving the MM in both grower and finisher phases rather than only in the finisher phase (86.0 vs. 79.1 and 180.2 vs. 169.2 g/bird/d, respectively; *P* < 0.01). At slaughtering, CON group presented higher BW than F6 and GF6, whereas F3 and GF3 presented intermediate values (2,541 vs. 2,454 vs. 2,412 vs. 2,445 vs. 2,384 g/bird for CON, F3, F6, GF3 and GF6, respectively; *P* < 0.01). Considering the overall trial period, DWG was higher in CON birds compared to GF6 ones, while the other groups did not present significant differences (59.6 vs. 58.7 vs. 57.6 vs. 57.5 vs. 56.0 g/bird/d for CON, F3, F6, GF3 and GF6, respectively; *P* < 0.05). DFI and mortality were not affected by the dietary treatments. GF6 group exhibited the highest FCR, which was significantly different from that of CON and F3 groups (1.785 vs. 1.810 vs. 1.834 vs. 1.886 vs. 1.934 g feed/g gain for CON, F3, F6, GF3, and GF6, respectively; *P* < 0.01). The contrast analysis revealed that MM administration reduced BW and DWG (2,541 vs. 2,424 g/bird and 59.6 vs. 57.4 g/bird/d for CON and MM; *P* < 0.01 and *P* < 0.05, respectively), while increased FCR (1.785 vs. 1.866 g feed/g gain, respectively; *P* < 0.05). No significant effect on performance traits emerged from the comparison of the 2 tested dosages, while MM inclusion during grower and finisher phases increased DFI and FCR in comparison to the use of MM in finisher phase only (108.3 vs. 105.8 g/bird/d and 1.910 vs. 1.822 g feed/g gain; *P* < 0.05 and *P* < 0.01, respectively).

**Table 2:** Growth performance of broiler chickens fed a conventional soybean-based diet (**CON**) or diets with different dosages of microalgae meal (30 or 60 g/kg) during finisher (F) or grower and finisher (**GF**) phase (n = 8 replicate pens/group).

Parameter	Experimental groups					SEM	*P-*value	Contrasts					
								Inclusion^2^		Dosage^3^		Duration^4^	
	CON	F3	F6	GF3	GF6			CON	MM	30 g/kg	60 g/kg	F	GF
	**Grower (14-28 d)**							**Grower (14-28 d)**					
**BW 28 d (g/bird)**	1,379 AB	1,392 AB	1,415 A	1,308 BC	1,284 C	12.3	<0.001	1,396 A	1,296 B	1,308	1,284	N/A	N/A
**DWG (g/bird/d)**^1^	69.6 A	70.6 A	72.2 A	64.6 B	62.5 B	0.79	<0.001	70.8 A	63.5 B	64.6	62.5	N/A	N/A
**DFI (g/bird/d)**^1^	113.4	111.0	113.4	114.5	113.0	0.59	0.47	112.6	113.8	114.5	113.0	N/A	N/A
**FCR (g feed/g gain)**^1^	1.636 B	1.574 B	1.573 B	1.777 A	1.811 A	0.02	<0.001	1,594 B	1.794 A	1.777	1.811	N/A	N/A
**Mortality (%)**	2.00	1.02	0.52	0.50	0.50	0.01	0.48	1.18	0.50	0.50	0.50	N/A	N/A
	**Finisher (29-41 d)**							**Finisher (29-41 d)**					
**BW 41 d (g/bird)**	2,541 A	2,454 AB	2,412 B	2,445 AB	2,384 B	15.6	<0.01	2,541 A	2,424 B	2,450	2,398	2,433	2,414
**DWG (g/bird/d)**^1^	89.5 A	81.8 B	76.4 C	87.5 A	84.6 AB	0.91	<0.001	89.5 A	82.5 B	84.6 A	80.5 B	79.1 B	86.0 A
**DFI (g/bird/d)**^1^	176.1 AB	171.5 AB	166.9 B	180.3 A	181.0 A	1.39	<0.01	176.1	174.9	175.9	174.0	169.2 B	180.2 A
**FCR (g feed/g gain)**^1^	1.971 B	2.102 AB	2.188 A	2.063 AB	2.143 A	0.02	<0.01	1.971 B	2.124 A	2.083 b	2.165 a	2.145	2.103
**Mortality (%)**	0.57	1.50	1.52	0.00	0.00	0.01	0.24	0.57	0.76	0.75	0.76	1.51 a	0.00 b
	**Overall trial (0-41 d)**							**Overall trial (0-41 d)**					
**BW 41 d (g/bird)**	2,541 A	2,454 AB	2,412 B	2,445 AB	2,384 B	15.6	<0.01	2,541 A	2,424 B	2,450	2,398	2,433	2,414
**DWG (g/bird/d)**^1^	59.6 a	58.7 ab	57.6 ab	57.5 ab	56.0 b	0.38	0.03	59.6 a	57.4 b	58.1	56.8	58.1	56.8
**DFI (g/bird/d)**^1^	106.2	106.1	105.5	108.4	108.1	0.50	0.24	106.2	107.1	107.3	106.8	105.8 b	108.3 a
**FCR (g feed/g gain)**^1^	1.785 B	1.810 B	1.834 AB	1.886 AB	1.934 A	0.02	<0.01	1.785 b	1.866 a	1.848	1.884	1.822 B	1.910 A
**Mortality (%)**	3.00	3.00	2.52	1.00	1.00	0.02	0.41	3.00	1.88	2.00	1.76	2.76	1.00

Abbreviations: BW, body weight; DWG, daily weight gain; DFI, daily feed intake; FCR, feed conversion ratio; SEM, standard error of the mean; CON, control diet; MM, microalgae meal diet; F, finisher phase; GF, grower and finisher phases; N/A, not applicable.

1 Corrected for mortality.

2 CON group vs. all groups receiving MM diets.

3 Groups fed diets with 30 g MM/kg feed vs. groups fed diets with 60 g MM/kg feed.

4 Groups fed MM diets during finisher phase vs. groups fed MM diets during grower and finisher phase. Means within a row not sharing a common superscript are significantly different (A, C: *P* < 0.01; a, b: *P* < 0.05).

### Slaughtering Measurements and Meat Quality Traits

**Figure 1:**
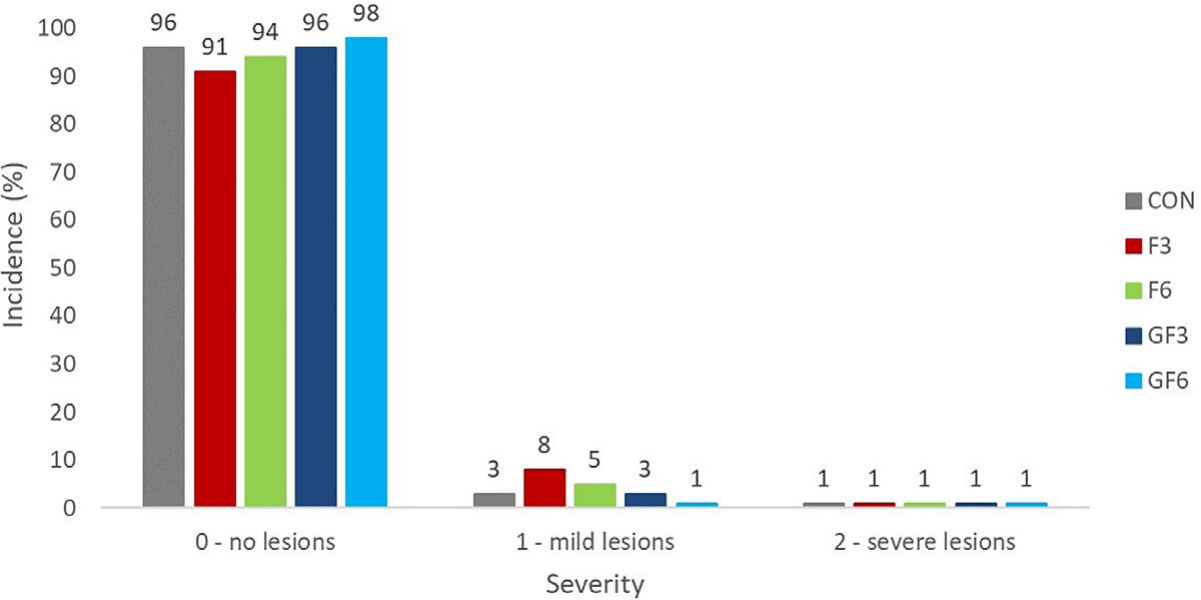
Incidence (%) and severity (0 – no lesions; 1 – mild lesions; 2 – severe lesions) of footpad dermatitis in 41-day-old broiler chickens fed a conventional soybean-based diet (**CON**) or diets with different dosages of microalgae meal (30 or 60 g/kg) during finisher (F) or grower and finisher (**GF**) phases. *n*: CON =152; F3 = 149; F6 = 170; GF3 = 164; GF6 = 168. Χ^2^*P*-value = 0.16.

Eviscerated carcass and breast yields, calculated on all processed birds (at least 150 birds/groups) but not statistically assessed as obtained on a group basis, were as follows: 65.3, 64.7, 63.8, 63.2, and 63.4% and 33.3, 32.9, 32.8, 33.3, and 32.5%, respectively for CON, F3, F6, GF3 and GF6 groups. There was no significant effect of the dietary treatments on the occurrence of FPD (Χ^2^ P-value = 0.16; Figure 1) as well as on the relative risk ratio of developing such condition (data not shown).

To as concern meat quality traits, the dietary incorporation of MM significantly reduced lightness while increased redness and yellowness in both raw and cooked breast meat (*P* < 0.001; Table 3). The effect of MM inclusion was particularly evident on meat yellowness (b*; raw meat: 6.77 vs. 12.7 vs. 17.0 vs. 17.2 vs. 20.1; cooked meat: 13.6 vs. 17.5 vs. 20.7 vs. 20.3 vs. 23.5 for CON, F3, F6, GF3 and GF6, respectively; *P* < 0.001). Breast meat yellow pigmentation increased as the dosage and the duration of MM administration increased (*P* < 0.01). Drip loss was significantly lower in GF6 group than in CON and F3 groups (1.87 vs. 1.87 vs. 1.75 vs. 1.61 vs. 1.54% for CON, F3, F6, GF3 and GF6, respectively; *P* < 0.05). The other quality traits of breast meat including pHu, cooking loss and shear force were not significantly influenced by the dietary treatments (Table 3).

**Table 3:** Technological properties of breast meat of broiler chickens fed a conventional soybean-based diet (**CON**) or diets with different dosages of microalgae meal (30 or 60 g/kg) during finisher (**F**) or grower and finisher (**GF**) phases (n = 15 breasts/group).

Parameter	Experimental groups					SEM	*P*-value	Contrasts					
								Inclusion^2^		Dosage^3^		Duration^4^	
	CON	F3	F6	GF3	GF6			CON	MM	30 g/kg	60 g/kg	F	GF
**pHu**	5.76	5.73	5.74	5.74	5.79	0.01	0.34	5.76	5.75	5.74	5.77	5.74	5.77
**Drip loss (%)**	1.87 a	1.87 a	1.75 ab	1.61 ab	1.54 b	0.04	<0.05	1.87	1.69	1.74	1.65	1.81	1.57
**Cooking loss (%)**	23.4	22.7	22.5	24.0	22.8	0.23	0.23	23.4	23.0	23.3	22.6	22.5	23.4
**Shear force (kg)**	2.1	2.2	2.1	2.1	1.9	0.04	0.16	2.1	2.1	2.2	2.0	2.2	2.0
**Lightness (L**^1^**) – raw meat**	57.9 a	56.6 ab	54.5 bc	54.3 c	52.6 c	0.31	<0.001	57.9 A	54.5 B	55.5 A	53.5 B	55.6 A	53.4 B
**Redness (a**^1^**) – raw meat**	1.67 b	1.65 b	2.69 a	2.72 a	3.13 a	0.10	<0.001	1.67 B	2.55 A	2.19 B	2.91 A	2.17 B	2.93 A
**Yellowness (b**^1^**) – raw meat**	6.77 d	12.7 c	17.0 b	17.2 b	20.1 a	0.59	<0.001	6.77 B	16.7 A	15.0 B	18.5 A	14.9 B	18.6 A
**Lightness (L**^1^**) – cooked meat**	83.7 a	83.4 ab	82.4 bc	82.4 bc	81.8 c	0.17	<0.001	83.7 A	82.5 B	82.9 a	82.1 b	82.9 a	82.1 b
**Redness (a**^1^**) – cooked meat**	2.15 B	2.44 B	3.10 A	3.14 A	3.55 A	0.09	<0.001	2.15 B	3.06 A	2.80 B	3.32 A	2.77 B	3.35 A
**Yellowness (b**^1^**) – cooked meat**	13.6 D	17.5 C	20.7 B	20.3 B	23.5 A	0.43	<0.001	13.6 B	20.5 A	18.9 B	22.1 A	19.1 b	21.9 a

Abbreviations: pHu, ultimate pH; SEM, standard error of the mean; CON, control diet; MM, microalgae meal diet; F, finisher phase; GF, grower and finisher phases.

1 Corrected for mortality.

2 CON group vs. all groups receiving MM diets.

3 Groups fed diets with 30 g MM/kg feed vs. groups fed diets with 60 g MM/kg feed.

4 Groups fed MM diets during finisher phase vs. groups fed MM diets during grower and finisher phase. Means within a row not sharing a common superscript are significantly different (A, D: *P* < 0.01; a, d: *P* < 0.05).

### Apparent Ileal Amino Acid Digestibility

The outcomes of the digestibility assay are reported in Table 4. Compared to CON group, F3 and F6 groups showed significantly lower apparent ileal digestibility for all analyzed amino acids. The reduction of digestibility coefficients in F3 and F6 groups ranged from 1.72 to 6.39% and from 4.40 to 18.0%, respectively, if compared to CON group. On average, F3 and F6 presented lower digestibility for both essential and non-essential amino acids (79.8 and 79.4% vs. 76.7 and 75.7% vs. 70.8 vs. 69.6%, respectively for CON, F3 and F6; *P* < 0.001).

**Table 4:** Apparent ileal amino acid digestibility in 41 d-old broilers fed soybean-based diets (CON) or diets with 30 or 60 g microalgae meal/kg feed during finisher phase (F3 and F6, respectively; n = 3 pools/group).

	Experimental groups								Var. (%)*	
AIAAD (%)	CON		F3		F6		SEM	*P*-value	F3 vs. CON	F6 vs. CON
**Methionine**	89.5	A	88.0	B	85.6	C	0.56	<0.001	−1.72	−4.40
**Cysteine**	71.0	A	67.8	B	58.2	C	1.87	<0.001	−4.43	−18.0
**Methionine +Cysteine**	82.5	A	80.6	B	76.5	C	0.80	<0.001	−2.28	−7.26
**Lysine**	83.3	A	80.9	B	77.1	C	0.89	<0.001	−2.83	−7.48
**Threonine**	72.8	A	70.4	B	63.4	C	1.38	<0.001	−3.24	−12.8
**Tryptophan**	74.3	A	69.5	B	61.4	C	1.82	<0.001	−6.39	−17.3
**Arginine**	87.1	A	83.3	B	79.8	C	1.02	<0.001	−4.35	−8.42
**Isoleucine**	79.2	A	75.0	B	68.4	C	1.52	<0.001	−5.20	−13.6
**Leucine**	80.9	A	77.8	B	72.4	C	1.21	<0.001	−3.76	−10.5
**Valine**	77.0	A	72.9	B	65.5	C	1.64	<0.001	−5.32	−15.0
**Histidine**	81.4	A	78.0	B	71.5	C	1.41	<0.001	−4.28	−12.2
**Phenylalanine**	83.3	A	80.3	B	74.8	C	1.21	<0.001	−3.67	−10.2
**Glycine**	73.3	A	69.8	B	62.4	C	1.56	<0.001	−4.74	−14.8
**Proline**	81.6	A	78.8	B	73.7	C	1.14	<0.001	−3.40	−9.76
**Serine**	76.4	A	72.6	B	65.4	C	1.56	<0.001	−5.01	−14.3
**Alanine**	77.1	A	73.9	B	67.0	C	1.45	<0.001	−4.22	−13.1
**Asparagine**	78.1	A	73.8	B	67.6	C	1.48	<0.001	−5.57	−13.4
**Glutamine**	86.1	A	82.8	B	78.4	C	1.08	<0.001	−3.85	−8.95
**Average EAA**	79.8	A	76.7	B	70.8	C	1.29	<0.001	−3.88	−11.3
**Average NEAA**	79.4	A	75.7	B	69.6	C	1.39	<0.001	−4.46	−12.3

Abbreviations: AIAAD, apparent ileal amino acid digestibility; EAA, essential amino acids; NEAA, non-essential amino acids.

⁎ Relative variation of F3 and F6 compared to CON (100%). Means within a row not sharing a common superscript are significantly different (A, C: *P* < 0.01).

### Plasma Metabolomics Profile

**Figure 2:**
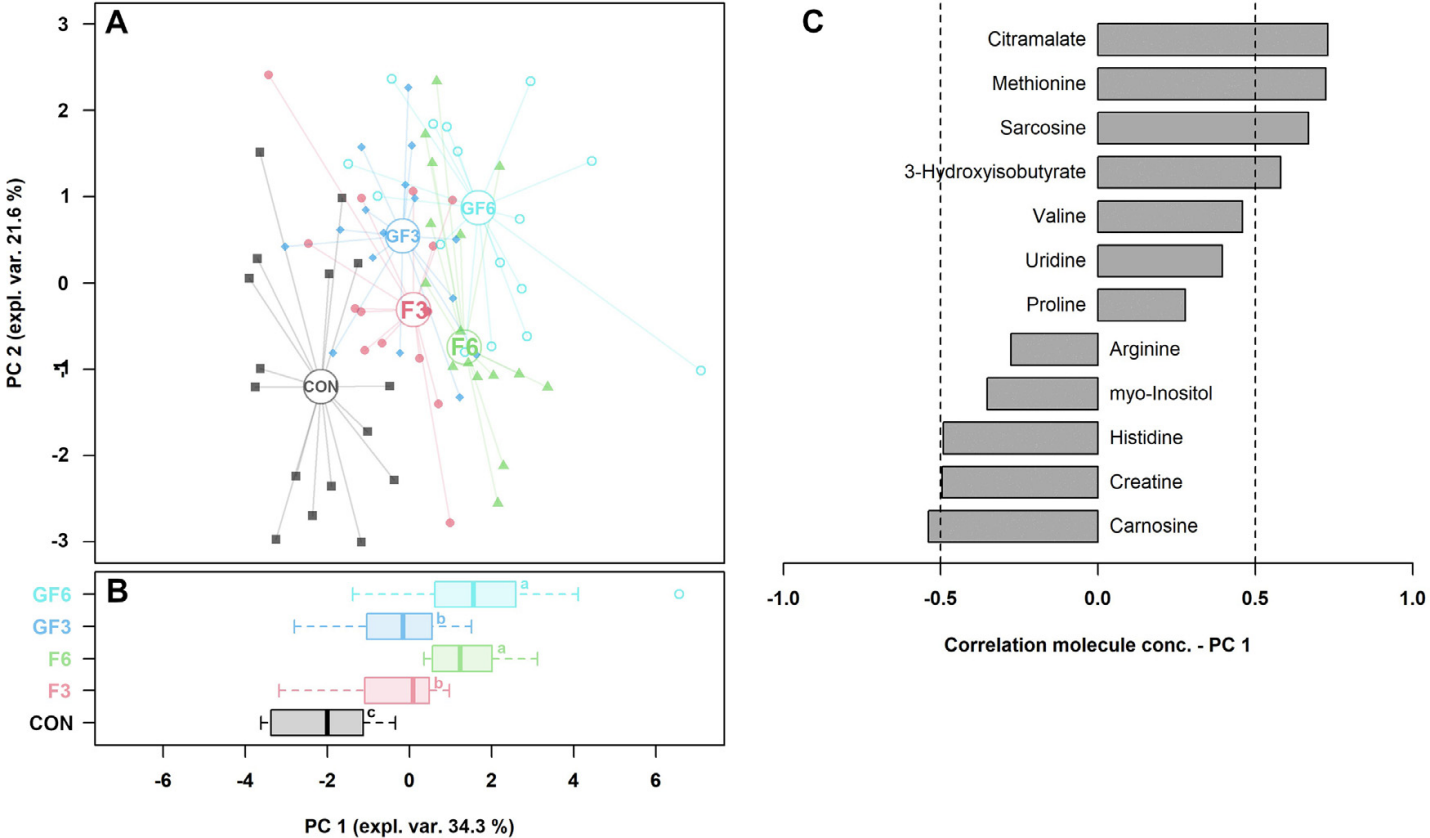
Robust principal component analysis model based on the concentration of plasma metabolites detected through ^1^H-NMR analysis in 41-day-old broiler chickens fed a conventional soybean-based diet (**CON**) or diets with different dosages of microalgae meal (30 or 60 g/kg) during finisher (**F**) or grower and finisher (**GF**) phases (n = 16 birds/group). (A) In the scoreplot, samples from CON, F3, F6, GF3, GF6 groups are shown with black squares, red circles, green triangles, blue rhombus and light blue empty circles, respectively. The wide, empty circles represent the means of the samples. (B) Boxplot depicting the position of the samples along the PC 1. (C) Loadingplot highlighting the correlation between the concentration of each metabolite and its importance over the PC1. Only significant correlations (*P* < 0.05) are shown. Abbreviations: PC, principal component; expl. Var, explained variance; conc., concentration. Means not sharing a common superscript are significantly different (a, c: *P* < 0.05).

A total of 60 plasma metabolites was identified through the ^1^H-NMR analysis. The concentration of 10 molecules, listed in Table 5, was significantly modified by the dietary inclusion of the *Arthrospira* meal. Specifically, the plasma levels of histidine (*P* < 0.001), creatine (*P* < 0.01), and arginine (*P* < 0.001) were reduced in GF6 compared to CON group. On the contrary, birds belonging to GF6 groups had greater plasma concentrations of citramalate (*P* < 0.001), sarcosine (*P* < 0.001), methionine (*P* < 0.001), uridine (*P* < 0.01), and 3-hydroxyisobutyrate (*P* < 0.01). The concentration of the remaining 50 metabolites is given in the Supplementary Materials. The first 2 components of the rPCA model described respectively 34.3 and 21.6% of the total variance among experimental groups (Figure 2). In the score plot (Figure 2, Panel A), the CON group tended to cluster separately from the groups receiving the MM into the diet. Moreover, the dietary dosage of MM significantly influenced the positioning of the experimental groups over the PC1. Indeed, while CON group was significantly different from all other experimental groups, those receiving MM shared a similar positioning according to the inclusion dosage.

**Table 5:** Concentration (mmol/L) of plasma metabolites identified through ^1^H-NMR analysis presenting significant differences in 41-day-old broilers fed soybean-based diets (CON) or diets with different dosages of microalgae meal (30 or 60 g/kg) during finisher (F) or grower and finisher (GF) phases (n = 16 birds/group).

Metabolite (mmol/L)	CON		F3		F6		GF3		GF6		SEM	*P*-value
**Histidine**	1.68E-01	A	1.44E-01	B	1.34E-01	BC	1.35E-01	BC	1.20E-01	C	2.66E-03	<0.001
**Citramalate**	8.67E-02	C	8.46E-02	C	1.06E-01	AB	9.26E-02	BC	1.10E-01	A	2.09E-03	<0.001
**Sarcosine**	4.83E-02	B	6.24E-02	B	7.84E-02	A	5.75E-02	B	8.38E-02	A	2.18E-03	<0.001
**Methionine**	2.33E-01	B	2.64E-01	AB	3.04E-01	A	2.80E-01	A	2.89E-01	A	5.63E-03	<0.001
**Arginine**	5.48E-01	A	4.61E-01	AB	3.73E-01	B	4.51E-01	AB	3.79E-01	B	1.56E-02	<0.01
**Uridine**	1.39E-02	B	2.35E-02	A	2.23E-02	A	2.03E-02	AB	2.45E-02	A	9.51E-04	<0.01
**myo-Inositol**	8.70E-01	AB	7.90E-01	B	8.14E-01	B	9.71E-01	A	8.04E-01	B	1.80E-02	<0.01
**Creatine**	1.23E-01	A	1.16E-01	AB	9.65E-02	AB	8.35E-02	AB	7.64E-02	B	4.95E-03	<0.01
**3-Hydroxyisobutyrate**	3.36E-02	B	3.92E-02	AB	4.23E-02	AB	3.77E-02	AB	4.63E-02	A	1.18E-03	<0.01
**Valine**	3.32E-01	b	3.59E-01	ab	3.75E-01	A	3.30E-01	b	3.54E-01	ab	4.73E-03	0.02

Means within a row not sharing a common superscript are significantly different (A, C: *P* < 0.01; a, b: *P* < 0.05).

## DISCUSSION

The evaluation of the animal response to the dietary administration of alternative protein sources such as microalgae represents a necessary step for their potential adoption in commercial feed formulation. In this study, fast-growing broilers were fed either finisher or grower and finisher diets in which *Arthrospira spp.* (Spirulina) meal was included at 30 or 60 g/kg as partial replacement for SBM. Overall, the results show that the dietary inclusion of MM had detrimental effects on the growth performance of broilers, as demonstrated by the lower BW and DWG and the higher FCR shown by birds fed diets with MM. This general trend was observed either considering the overall rearing cycle (0–41 d) or each single feeding phase. The reduction of growth performance was particularly evident in GF6 groups, whereas F3 birds presented comparable BW and FCR to CON, even though the numerical differences between these groups can however be remarkable. No significant effect was observed on DFI, indicating that MM administration had no negative effects on voluntary feed consumption and overall feed acceptability, at least at the dosages tested in the present study. Taken together, these results suggest that 60 g MM/kg feed can significantly depress the growth performance of broiler chickens, whereas 30 g/kg from 29 to 41 d could be partially tolerated without excessive detriments of productive traits in the overall rearing cycle. Although the available literature concerning the use of *Arthrospira spp*. - Spirulina in broiler chicken feeding is quite abundant, the number of studies focusing on its use as major source of dietary proteins is still limited. The early results reported by Ross and Dominy (1990) shows that the dietary inclusion of 30 or 60 g Spirulina *per* kg of feed for 41 d did not affect the growth performance of Hubbard x Hubbard chickens. Similarly, feeding diets with 40 or 80 g Spirulina meal/kg from 21 to 37 d of age exerted no significant effects on body weight of Arbor Acres broilers (Toyomizu et al., 2001). Despite of the differences in the experimental methodologies and designs among recent studies dealing with this topic (e.g., genotype, slaughter age, farming conditions, etc.), it can be observed that Spirulina inclusion up to 10 to 20 g/kg generally provide either positive or no effects on the growth performance of chickens (Mirzaie et al., 2018; Park et al., 2018; Sugiharto et al., 2018; Khan et al., 2020). Our results are partially in line with the conclusion of Coudert et al. (2020) who stated that dietary dosages of microalgae around 20 g/kg could be considered as suitable to maintain acceptable growth performance in broiler chickens. In our study, growth performances seem to be particularly impaired once the birds were switched from the SBM-based diet to the MM-based one (i.e., as occurred to GF3 and GF6 in the grower phase and to F3 and F6 in the finisher one, respectively), which might be due to the different composition and digestibility of the experimental diets. Unexpectedly, the duration of MM administration significantly affected DFI in the overall trial period, with birds receiving the MM during both grower and finisher phase (14–41 d) that have consumed more feed than those fed MM during the finisher phase only (29–41 d). However, such increase in feed intake (whose reasons need to be further investigated) was not associated with a higher DWG, resulting in poorer feed efficiency.

The occurrence of FPD, which is considered an important welfare indicator for broiler chickens (Shepherd and Fairchild, 2010), as well as the risk of developing such lesions were not affected by the dietary treatments. It should be considered that the overall occurrence of this lesion was very low in our study, which indicates optimal management of the litter and environmental conditions. In general, the absence of adverse effects on the incidence and severity of FPD in response to the dietary utilization of an alternative, protein-rich ingredient such as MM can be regarded as a positive outcome. Indeed, it is widely known that the quality of dietary protein can affect nitrogen excretion as well as water consumption and, in turn, litter moisture, which are key factors in the onset of FPD (Shepherd and Fairchild, 2010). To the best of our knowledge, only Mullenix et al. (2022) conducted this type of evaluation on broilers fed diets supplemented with MM. The authors asserted that the incorporation of 10 g Spirulina / kg feed into low crude protein diets from 15 to 35 or 37 d diminished the average FPD scores in male chickens if compared to a standard crude protein diet, but not if compared to the same low crude protein diet without Spirulina.

**Figure 3:**
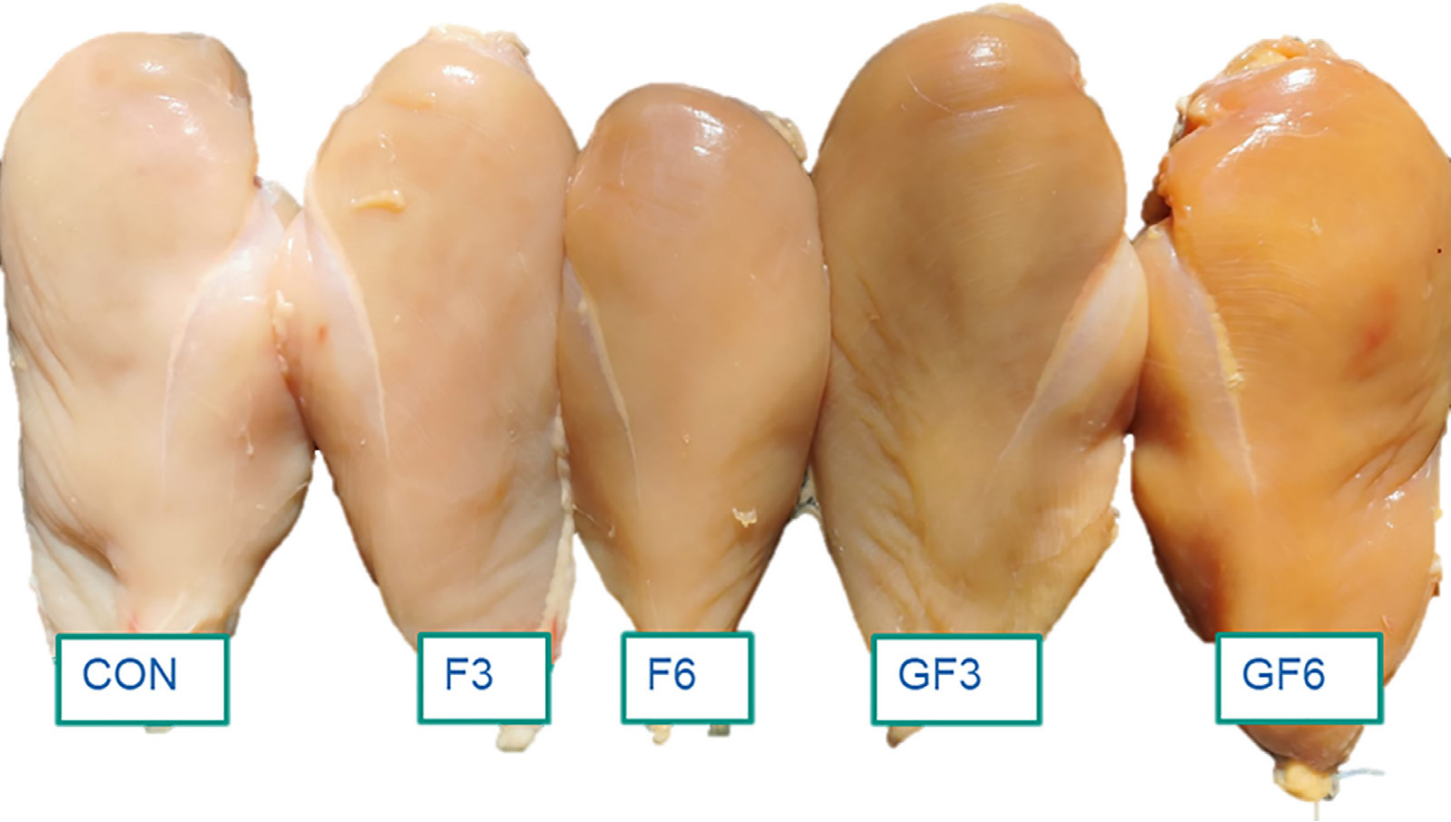
Appearance of breast fillets of 41-day-old broiler chickens fed a conventional soybean-based diet (**CON**) or diets with different dosages of microalgae meal (30 or 60 g/kg) during finisher (**F**) or grower and finisher (**GF**) phases.

As far as breast meat quality is concerned, it can be concluded that the tested diets did not substantially modify most of the technological traits considered in this study. The most relevant effect of the dietary treatments was, as expected, on the color profile. Indeed, for both raw and cooked meat, the administration of MM diets increased meat yellow pigmentation, with appearance variations of raw meat that were detectable not only instrumentally but also visually (Figure 3). Overall, this outcome is in line with previous studies (Toyomizu et al., 2001; Pestana et al., 2020; Mullenix et al., 2022), which indicated that *Arthrospira* inclusion can increase chicken meat color. Conversely, Park et al. (2018) showed that dietary dosages up to 10 g/kg did not modify the color profile of breast meat. Spirulina is a natural source of pigments including carotenoids (total: 0.28–2.23 mg/g; Park et al., 2018), such as β-carotene, zeaxanthin and lutein, as well as phycobiliproteins like c-phycocyanin (Ranga Rao et al., 2010; Park et al., 2018). According to Kalia and Lei (2022), the higher redness value of poultry meat observed in response to the dietary MM administration might be related to the increment of myoglobin levels induced by the high iron and mineral content of some microalgae. Similarly, the deposition of zeaxanthin in the muscle tissue is retained to be the main reason behind the increased meat yellowness in broiler fed MM-enriched diets (Kalia and Lei, 2022). Taken together, the inclusion of MM in broiler diets, even at relatively low dosages and for a limited period of the rearing cycle, confirmed to be an interesting approach to increase skin and meat yellowness. This is particularly relevant in the light of the recent limitation on the use of synthetic yellow pigments in Europe (Regulation 2020/1400) and, in general, of the growing interest of consumers in purchasing products from animal fed only natural ingredients especially produced using alternative farming systems (i.e., free range, organic). Finally, if compared to the CON group, drip loss was improved by the dietary administration of 60 g MM/kg during both grower and finisher phases even though pH value exhibited no remarkable variation. Previously, Park et al. (2018) observed a reduction in drip loss after 7 d of refrigerated storage when Spirulina was included at a dosage of 10 g/kg. Although further studies on this topic are needed, it could be speculated that the improvements in meat water holding capacity might be related to the antioxidant effects of the bioactive molecules present in MM, notably the aforementioned pigments, which could have reduced oxidative lipid and protein damages on cell membrane occurring during post-mortem time thus enhancing their ability to retain water during storage.

A significant reduction of ileal AA digestibility was observed in broilers fed on diets presenting MM, particularly at the highest tested dosage. Such negative outcome could be accounted as one of the factors responsible for the impairment of growth performance in MM-fed groups. Previously, Tavernari et al. (2018) determined the AA digestibility coefficients of *Arthrospira platensis* by feeding Cobb 500 male broilers a basal diet with 200 g Spirulina/kg feed for 10 d. Results showed that, at 24 d of age, the standardized ileal digestibility coefficients were 0.80 for essential and 0.78 for non-essential AA (apparent digestibility coefficients = 0.74 and 0.71, respectively), which are generally in line with our observations even considering the methodological differences between the studies as the current one did not correct for basal AA endogenous loss. Evans et al. (2015), testing Spirulina inclusion levels up to 160 g/kg from 3 to 21 d, found either similar or better apparent ileal AA digestibility compared to a conventional corn-SBM diet, even though higher dosages (i.e., 210 g/kg) depressed such parameter. Finally, Park et al. (2018) stated that the apparent total tract digestibility of nitrogen linearly increased when diets supplemented with *Arthrospira spp.* (from 0 to 10 g/kg) were provided over a 35-d rearing cycle. Being a cyanobacterium, *Arthrospira spp.* has a gram-negative cell wall composed of peptidoglycan with no cellulosic compounds in its structure (Machado et al., 2022). Although it could be considered more fragile if compared to that of other microalgae (e.g., *Chlorella vulgaris* and *Haematococcus lacustris*), the peptidoglycan barrier could interfere with the digestibility, bio-accessibility and bio-availability of Spirulina proteins in monogastric species such as poultry (Spinola et al., 2022). Moreover, elevated dosages of Spirulina (i.e., 150 g/kg) were associated with an increase in digesta viscosity potentially induced by the resistance of microalgae proteins to endogenous peptidases, which could remarkably limit nutrient digestibility (Pestana et al., 2020; Spinola et al., 2022). Within this context, the hypothesis of Pestana et al. (2020), *viz*., using specific exogenous enzymes to support nutrient digestibility and growth performance of birds fed on diets containing MM, merits further investigations.

The plasma metabolome has been substantially modified by the use of *Arthrospira spp.* as alternative protein source. Focusing the discussion on the most meaningful metabolomics changes, it emerged that the administration of MM reduced the plasma concentration of histidine as well as that of the histidine-containing dipeptide carnosine (*P* = 0.05; Supplementary Materials) compared to the CON group. Being an essential amino acid, the most likely explanation for the reduced plasmatic histidine levels could be its lower intestinal digestibility in MM-fed groups. In turn, the drop of carnosine content is not surprising being the plasma content of these 2 molecules characterized by a strong positive correlation (Lackner et al., 2021). Previous metabolomics investigations performed by our research group revealed that greater plasma histidine concentrations were associated with improvements of growth performance and feed efficiency in broiler chickens (Zampiga et al., 2018; Brugaletta et al., 2023). On the other hand, Kai et al. (2015) pointed out that providing a histidine-deficient diet can impair body weight gain and breast muscle development. In addition, plasma histidine concentration was reported to be positively correlated with *P. major* weight (Baeza et al., 2015), which is in line with the lower breast yield observed in GF6 birds (-0.8% compared to CON). Analogously, the reduction of intestinal digestibility could be the culprit of the lower plasma concentration of arginine detected in birds fed MM at the dosage of 60 g/kg. Arginine is an essential amino acid for chickens, with implications on a plethora of physiological and immunological traits (Khajali and Wideman, 2010; Fouad et al., 2012). Furthermore, it is considered as a potent secretagogue for anabolic hormones such as growth hormone, insulin and insulin-like growth factor-1, which play a key role in supporting the metabolic functions and thus the growth performance of broilers (Fernandes and Murakami, 2010). Increased plasma levels of arginine have been associated with enhanced feed efficiency and breast muscle yield in broiler chickens (Zampiga et al., 2018; Brugaletta et al., 2023). Arginine is also involved in the hepatic synthesis of creatine, the precursor of phosphocreatine that is known to act as a high-energy phosphate reserve for the regeneration of ATP in muscles and other tissues (Wyss and Kaddurah-Daouk, 2000; Oviedo-Rondón and Córdova-Noboa, 2020). Several works highlighted the paramount importance of creatine or guanidinoacetate (i.e., the creatine precursor used as feed additive in poultry diets) on metabolic functions and productive traits of modern broilers (Oviedo-Rondón and Córdova-Noboa, 2020; Portocarero and Braun, 2021). As the plasma levels of creatine were significantly lower in GF6 group compared to CON one (ca. -37%), it could be hypothesized that the limited creatine availability could have contributed to the reduced growth performance of that group. Taken together, the variations in the plasma levels of some metabolites that take part in relevant metabolic processes, such as energy metabolism and homeostasis as well as protein synthesis, could provide some clues for the worsening of productive performance in MM-fed groups, particularly in the GF6 one.

In conclusion, the dietary administration of *Arthrospira spp.* meal at 60 g/kg during the grower and finisher phases as partial replacement for SBM impaired the growth performance of broiler chickens, whereas a lower inclusion dosage (30 g/kg) during the finisher phase can be partially tolerated. No significant effect of the dietary treatments was observed on the occurrence of FPD as well as on the risk of developing such lesions. The dietary utilization of MM increased breast meat yellowness, whereas other technological traits were not substantially affected. The negative performance results of MM-fed groups were associated with a generalized reduction of intestinal amino acid digestibility, which was particularly evident in the groups receiving the MM at the highest tested dosage. Finally, the ^1^H-NMR analysis revealed significant variations in the plasma concentration of some metabolites involved in crucial metabolic processes such as energy metabolism and homeostasis as well as protein synthesis, which could explain, at the molecular level, the physiological reasons behind the worsening of growth performance in broilers fed on diets containing MM. Further investigations could be helpful to clarify whether feed technological processing (e.g. pelleting) and/or the use of exogenous enzymes could provide benefits when MM is incorporated into broiler diets.

## DISCLOSURES

The authors declare no conflicts of interest.

## Appendix Supplementary materials

Image, application 1

## Footnotes

Supplementary material associated with this article can be found in the online version at doi:10.1016/j.psj.2024.103856.
