# Supplementary material for: Partial substitution of soybean meal with microalgae meal (Arthrospira spp. – Spirulina) in grower and finisher diets for broiler chickens: implications on performance parameters, footpad dermatitis occurrence, breast meat quality traits, amino acid digestibility and plasma metabolomics profile
Source: Poult Sci. 2024 May 21;103(8):103856. doi: 10.1016/j.psj.2024.103856 (PMC11253657; doi:10.1016/j.psj.2024.103856)
Supplement: Supplementary file 1 [file mmc1.docx]

**SUPPLEMENTARY MATERIALS**

**Article Title:** Partial substitution of soybean meal with microalgae meal (*Arthrospira spp.* – Spirulina) in grower and finisher diets for broiler chickens: implications on performance parameters, footpad dermatitis occurrence, breast meat quality traits, amino acid digestibility and plasma metabolomics profile

**Authors:** Marco Zampiga ^*^, Luca Laghi ^*^, Francesca Soglia ^*^, Raffaela Piscitelli ^*^, Jonathan Dayan *^†^, Massimiliano Petracci ^*^, Alessio Bonaldo ^‡^, Federico Sirri ^*,1^

* Department of Agricultural and Food Sciences, Alma Mater Studiorum - University of Bologna, Via del Florio 2, 40064, Ozzano dell’Emilia, Bologna, Italy

^†^ Department of Animal Science, The Robert H. Smith Faculty of Agriculture, Food and Environment, The Hebrew University of Jerusalem, Rehovot 7610001, Israel

^‡^ Department of Veterinary Medical Sciences, Alma Mater Studiorum - University of Bologna, Via Tolara di Sopra 50, 40064, Ozzano Emilia, Bologna, Italy

**Supplementary Figure 1. Chemical composition (A), amino acid profile (B) and fatty acid profile (C) of the microalgae meal tested in the study.**

A: Chemical composition (results are expressed as g/kg of microalgae meal)


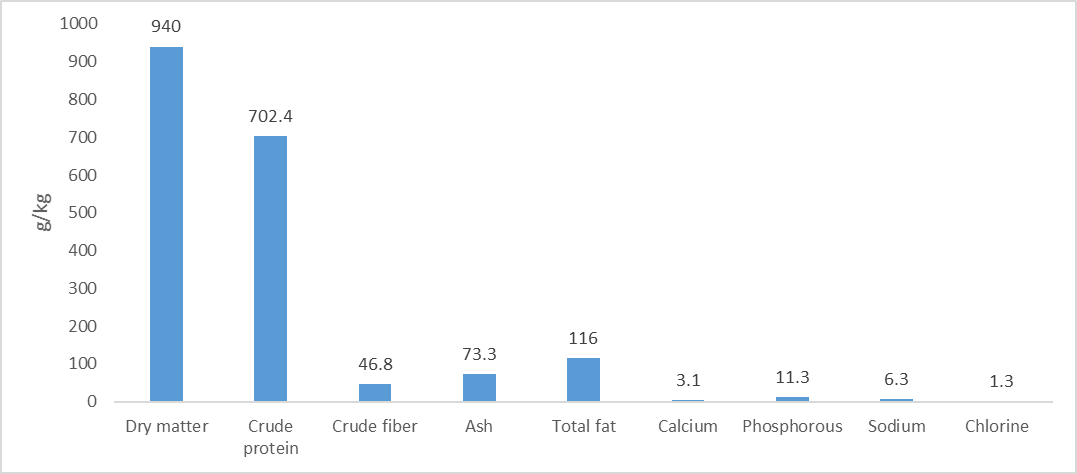


B: Amino acid profile (results are expressed as g/kg of microalgae meal)


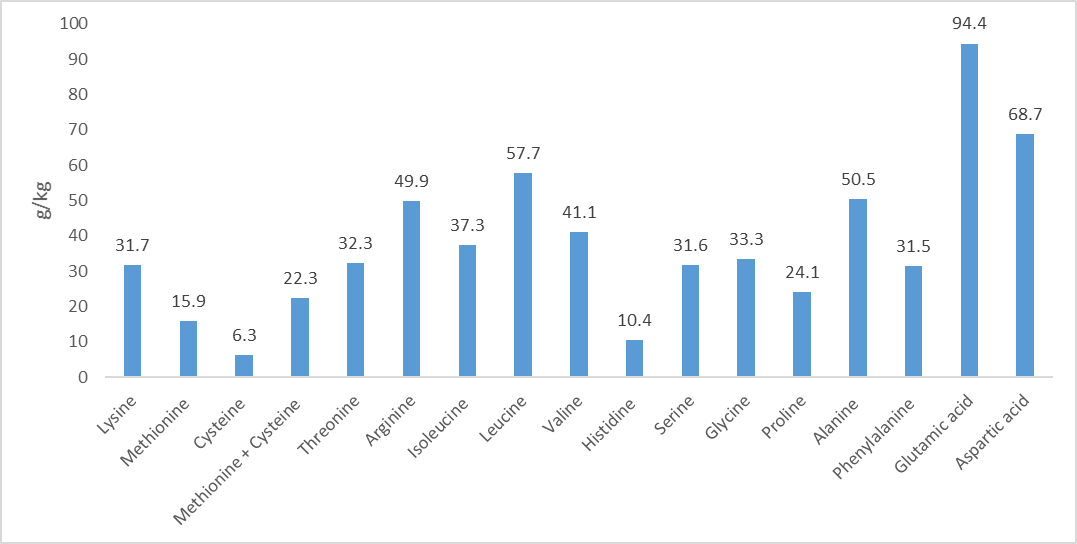


C: Fatty acid profile (results are expressed as percentage)


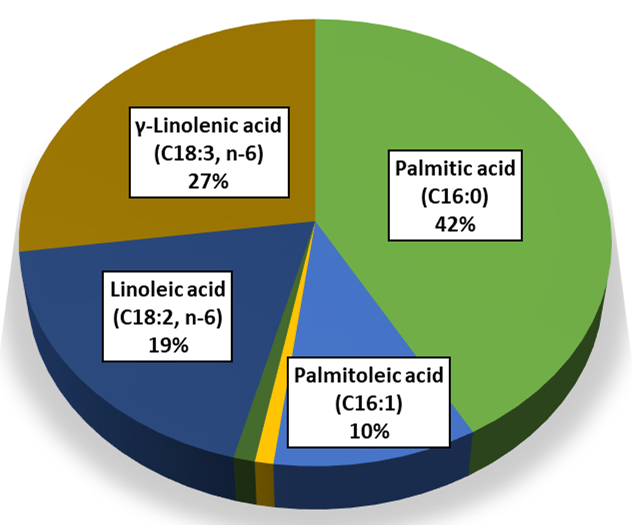


**Supplementary Table 1. Growth performance of the groups during the starter phase (0 – 12 d).**

|  | **CON** | **F3** | **F6** | **GF3** | **GF6** | **SEM** | ***P*-value** |
| --- | --- | --- | --- | --- | --- | --- | --- |
| **Chick body weight (g)** | 37.7 | 37.3 | 38.0 | 37.5 | 38.0 | 0.11 | 0.21 |
| **Body weight (g)** | 325 | 330 | 331 | 330 | 333 | 2.46 | 0.90 |
| **Daily weight gain (g/bird/d)*** | 22.0 | 22.5 | 22.4 | 22.5 | 22.6 | 0.20 | 0.91 |
| **Daily feed intake (g/bird/d)*** | 36.0 | 36.4 | 36.2 | 36.2 | 36.4 | 0.17 | 0.95 |
| **Feed intake (kg/bird)*** | 0.467 | 0.473 | 0.470 | 0.471 | 0.473 | 0.002 | 0.95 |
| **Feed conversion ratio*** | 1.634 | 1.622 | 1.617 | 1.610 | 1.611 | 0.01 | 0.97 |
| **Mortality (%)** | 0.50 | 0.50 | 0.50 | 0.50 | 0.50 | 0.01 | 0.99 |

Abbreviations: SEM = standard error of the mean

*: Corrected for mortality.

**Supplementary Table 2. Concentration (mmol/L) of metabolites, detected through the ^1^H-NMR analysis, showing no significant difference (P>0.05) in the plasma of 41-d-old broilers fed soybean-based diets (CON) or diets with different dosages of microalgae meal (30 or 60 g/kg) during finisher (F) or grower and finisher (GF) phases (n = 16 birds/group).**

| **Molecule (mmol/L)** | **CON** | **F3** | **F6** | **GF3** | **GF6** | **SEM** | ***P*-value** |
| --- | --- | --- | --- | --- | --- | --- | --- |
| Carnosine | 7.31E-02 | 7.37E-02 | 5.14E-02 | 5.83E-02 | 5.58E-02 | 2.87E-03 | 0.05 |
| Alanine | 2.16E+00 | 2.31E+00 | 2.47E+00 | 2.20E+00 | 2.25E+00 | 3.63E-02 | 0.05 |
| Proline | 5.10E-01 | 5.13E-01 | 5.50E-01 | 4.77E-01 | 5.00E-01 | 8.08E-03 | 0.07 |
| Threonine | 1.23E+00 | 1.15E+00 | 1.24E+00 | 1.29E+00 | 1.39E+00 | 2.66E-02 | 0.07 |
| Leucine | 3.54E-01 | 3.93E-01 | 4.00E-01 | 3.78E-01 | 3.76E-01 | 5.61E-03 | 0.09 |
| Tyrosine | 4.13E-01 | 4.61E-01 | 5.07E-01 | 4.18E-01 | 4.22E-01 | 1.27E-02 | 0.10 |
| Isopropanol | 1.49E-02 | 1.86E-02 | 1.49E-02 | 1.70E-02 | 1.27E-02 | 7.27E-04 | 0.10 |
| Ascorbate | 1.33E-01 | 1.23E-01 | 1.06E-01 | 1.33E-01 | 1.19E-01 | 3.85E-03 | 0.14 |
| Isoleucine | 1.67E-01 | 1.68E-01 | 1.75E-01 | 1.58E-01 | 1.67E-01 | 2.07E-03 | 0.15 |
| Glycerol | 2.50E-01 | 2.58E-01 | 2.25E-01 | 2.37E-01 | 2.72E-01 | 6.69E-03 | 0.20 |
| 2,3-butanediol | 1.34E-01 | 1.64E-01 | 1.69E-01 | 1.56E-01 | 1.93E-01 | 7.81E-03 | 0.21 |
| Betaine | 1.42E+00 | 1.37E+00 | 1.40E+00 | 1.43E+00 | 1.25E+00 | 2.62E-02 | 0.22 |
| Dimethyl-Sulfone | 1.09E-01 | 1.24E-01 | 9.58E-02 | 1.50E-01 | 9.74E-02 | 8.46E-03 | 0.23 |
| Pyroglutamate | 1.38E-01 | 1.51E-01 | 1.28E-01 | 1.58E-01 | 1.45E-01 | 4.67E-03 | 0.30 |
| 2-Hydroxybutyrate | 3.17E-02 | 3.25E-02 | 3.17E-02 | 4.17E-02 | 4.15E-02 | 2.14E-03 | 0.30 |
| Dimethylamine | 7.12E-03 | 5.46E-03 | 4.45E-03 | 1.21E-02 | 6.25E-03 | 1.21E-03 | 0.30 |
| 2-Aminobutyrate | 4.96E-02 | 6.53E-02 | 5.99E-02 | 6.86E-02 | 6.28E-02 | 2.94E-03 | 0.31 |
| Trimethylamine-N-oxide | 3.33E-02 | 2.90E-02 | 2.86E-02 | 2.58E-02 | 2.66E-02 | 1.22E-03 | 0.33 |
| Methanol | 8.28E-02 | 7.50E-02 | 8.80E-02 | 8.84E-02 | 9.33E-02 | 2.95E-03 | 0.36 |
| Pyruvate | 4.08E-01 | 4.51E-01 | 4.12E-01 | 4.33E-01 | 4.38E-01 | 7.57E-03 | 0.36 |
| 3-Hydroxybutyrate | 7.51E-01 | 7.07E-01 | 7.72E-01 | 9.17E-01 | 7.78E-01 | 3.43E-02 | 0.38 |
| 2-Oxoglutarate | 2.14E-01 | 2.07E-01 | 2.22E-01 | 2.33E-01 | 2.25E-01 | 4.29E-03 | 0.38 |
| Aspartate | 1.55E-01 | 1.55E-01 | 1.30E-01 | 1.41E-01 | 1.32E-01 | 5.54E-03 | 0.48 |
| Fumarate | 2.05E-02 | 2.14E-02 | 1.99E-02 | 2.19E-02 | 2.18E-02 | 4.19E-04 | 0.50 |
| Hypoxanthine | 1.49E-02 | 1.70E-02 | 1.57E-02 | 1.65E-02 | 1.46E-02 | 5.10E-04 | 0.51 |
| Arabinose | 1.20E-01 | 1.16E-01 | 1.19E-01 | 1.17E-01 | 1.10E-01 | 1.85E-03 | 0.53 |
| Lactate | 2.03E+01 | 2.13E+01 | 1.86E+01 | 2.02E+01 | 2.00E+01 | 4.75E-01 | 0.54 |
| N,N-Dimethylglycine | 1.93E-01 | 1.91E-01 | 1.77E-01 | 1.82E-01 | 1.83E-01 | 3.35E-03 | 0.57 |
| Beta-Alanine | 1.26E-01 | 1.50E-01 | 1.53E-01 | 1.48E-01 | 1.53E-01 | 6.19E-03 | 0.61 |
| Methylmalonate | 6.00E-02 | 4.29E-02 | 4.51E-02 | 4.57E-02 | 4.63E-02 | 3.71E-03 | 0.61 |
| Glutamine | 2.44E+00 | 2.53E+00 | 2.61E+00 | 2.39E+00 | 2.53E+00 | 4.62E-02 | 0.62 |
| Mannose | 6.83E-02 | 7.44E-02 | 6.17E-02 | 6.28E-02 | 6.07E-02 | 3.13E-03 | 0.62 |
| Anserine | 4.45E-02 | 4.36E-02 | 4.49E-02 | 4.55E-02 | 4.03E-02 | 1.15E-03 | 0.63 |
| 3-Methyl-2-oxovalerate | 1.55E-02 | 1.50E-02 | 1.51E-02 | 1.60E-02 | 1.47E-02 | 3.00E-04 | 0.68 |
| Acetate | 7.23E-02 | 7.05E-02 | 7.61E-02 | 5.79E-02 | 6.59E-02 | 4.16E-03 | 0.70 |
| Choline | 8.43E-02 | 7.95E-02 | 7.91E-02 | 8.04E-02 | 7.81E-02 | 1.49E-03 | 0.74 |
| Serine | 1.36E+00 | 1.36E+00 | 1.47E+00 | 1.41E+00 | 1.44E+00 | 3.16E-02 | 0.75 |
| Glucose | 2.37E+01 | 2.29E+01 | 2.36E+01 | 2.36E+01 | 2.30E+01 | 2.30E-01 | 0.76 |
| Succinate | 1.19E-01 | 1.22E-01 | 1.10E-01 | 1.11E-01 | 1.17E-01 | 3.31E-03 | 0.76 |
| Ethanol | 1.08E-01 | 8.34E-02 | 8.16E-02 | 8.57E-02 | 8.95E-02 | 7.16E-03 | 0.77 |
| Acetone | 3.39E-02 | 3.63E-02 | 3.58E-02 | 3.71E-02 | 3.42E-02 | 1.01E-03 | 0.83 |
| Alpha-ketoisovaleric acid | 6.10E-03 | 6.64E-03 | 6.47E-03 | 6.07E-03 | 6.01E-03 | 2.10E-04 | 0.85 |
| Asparagine | 3.86E-01 | 3.68E-01 | 4.01E-01 | 3.75E-01 | 3.92E-01 | 1.14E-02 | 0.90 |
| Theophylline | 4.80E-03 | 4.68E-03 | 4.45E-03 | 4.57E-03 | 4.70E-03 | 1.15E-04 | 0.90 |
| Formate | 2.00E-01 | 1.86E-01 | 2.02E-01 | 1.95E-01 | 2.05E-01 | 6.58E-03 | 0.91 |
| Lysine | 1.46E-01 | 1.35E-01 | 1.41E-01 | 1.27E-01 | 1.51E-01 | 8.51E-03 | 0.91 |
| Phenylalanine | 2.20E-01 | 2.22E-01 | 2.16E-01 | 2.18E-01 | 2.19E-01 | 2.42E-03 | 0.96 |
| Glycine | 1.28E+00 | 1.31E+00 | 1.31E+00 | 1.27E+00 | 1.32E+00 | 2.50E-02 | 0.97 |
| trans-4-Hydroxy-L-proline | 1.65E-01 | 1.69E-01 | 1.67E-01 | 1.70E-01 | 1.73E-01 | 4.83E-03 | 0.99 |
| Citrate | 5.44E-01 | 5.52E-01 | 5.50E-01 | 5.45E-01 | 5.47E-01 | 9.82E-03 | 0.99 |

Abbreviations: SEM = standard error of the mean
